# Sepsis in two hospitals in Rwanda: A retrospective cohort study of presentation, management, outcomes, and predictors of mortality

**DOI:** 10.1371/journal.pone.0251321

**Published:** 2021-05-26

**Authors:** Dennis A. Hopkinson, Jean Paul Mvukiyehe, Sudha P. Jayaraman, Aamer A. Syed, Myles S. Dworkin, Willy Mucyo, Thierry Cyuzuzo, Anne Tuyizere, Christian Mukwesi, Gaston Nyirigira, Paulin R. Banguti, Elisabeth D. Riviello

**Affiliations:** 1 Department of Internal Medicine, Virginia Commonwealth University, Richmond, Virginia, United States of America; 2 Department of Anesthesia, University of Rwanda College of Medicine and Health Sciences, Kigali, Rwanda; 3 Department of Surgery, Virginia Commonwealth University, Richmond, Virginia, United States of America; 4 Sidney Kimmel Medical College, Thomas Jefferson University, Philadelphia, Pennsylvania, United States of America; 5 King Faisal Hospital Kigali, Kigali, Rwanda; 6 University of Rwanda College of Medicine and Health Sciences, Kigali, Rwanda; 7 Rwanda Military Hospital, Kigali, Rwanda; 8 University Teaching Hospital of Butare, Butare, Rwanda; 9 Division of Pulmonary, Critical Care, and Sleep Medicine, Beth Israel Deaconess Medical Center and Harvard Medical School, Boston, Massachusetts, United States of America; Wayne State University, UNITED STATES

## Abstract

**Purpose:**

Few studies have assessed the presentation, management, and outcomes of sepsis in low-income countries (LICs). We sought to characterize these aspects of sepsis and to assess mortality predictors in sepsis in two referral hospitals in Rwanda.

**Materials and methods:**

This was a retrospective cohort study in two public academic referral hospitals in Rwanda. Data was abstracted from paper medical records of adult patients who met our criteria for sepsis.

**Results:**

Of the 181 subjects who met eligibility criteria, 111 (61.3%) met our criteria for sepsis without shock and 70 (38.7%) met our criteria for septic shock. Thirty-five subjects (19.3%) were known to be HIV positive. The vast majority of septic patients (92.7%) received intravenous fluid therapy (median = 1.0 L within 8 hours), and 94.0% received antimicrobials. Vasopressors were administered to 32.0% of the cohort and 46.4% received mechanical ventilation. In-hospital mortality for all patients with sepsis was 51.4%, and it was 82.9% for those with septic shock. Baseline characteristic mortality predictors were respiratory rate, Glasgow Coma Scale score, and known HIV seropositivity.

**Conclusions:**

Septic patients in two public tertiary referral hospitals in Rwanda are young (median age = 40, IQR = 29, 59) and experience high rates of mortality. Predictors of mortality included baseline clinical characteristics and HIV seropositivity status. The majority of subjects were treated with intravenous fluids and antimicrobials. Further work is needed to understand clinical and management factors that may help improve mortality in septic patients in LICs.

## Introduction

Sepsis is a dysregulated host response to infection that results in life-threatening organ dysfunction [1]. Based on data from the Global Burden of Diseases, Injuries, and Risk Factors Study (GBD) 2017, an estimated 48.9 million people worldwide experienced sepsis in 2017, with 11.0 million deaths [2]. This burden is particularly high in low-income countries (LICs), with an estimated 16.7 million cases of sepsis occurring annually in sub-Saharan Africa [2]. A meta-analysis of studies in sub-Saharan Africa demonstrated a sepsis mortality rate of 19% and a severe sepsis mortality rate of 39% [3]. In Rwanda, the mortality in two intensive care units was shown to be 64.4% for sepsis and 82.1% for septic shock [4].

Although these data demonstrate a high incidence and mortality from sepsis in LICs, few studies on sepsis presentation, management, outcomes, and predictors of mortality in LICs have been reported. In a study assessing critically ill patients with suspected infection in a public tertiary referral center in Kenya, the majority (65.1%) were suffering from respiratory infections and the most common comorbidity was diabetes mellitus (27.3%) [5]. In a survey of critical care providers at a large tertiary center in Kenya, respondents indicated the most common sources of infection in sepsis were respiratory and intra-abdominal, the most frequently used antibiotics were ceftriaxone and metronidazole, and 43% of respondents ordered blood cultures on suspicion of sepsis regularly [6]. In a private hospital in rural Uganda, a study in 20 adults and 31 children with sepsis found that the most common comorbidity in adults was HIV (30%); greater than 80% of patients received an antibiotic; and half of all patients with an elevated lactate received intravenous fluid resuscitation [7]. In a referral hospital in the capital city of Haiti, the most common sources of infection in patients with sepsis were lung and intra-abdominal [8]. Management at this facility consisted of intravenous fluid resuscitation in 80% of patients with severe sepsis, and 54.6% of patients with severe sepsis received antimicrobials within 24 hours, with the most commonly used antimicrobials being ceftriaxone and chloroquine.

Mortality from sepsis in most of these observational studies at large referral centers ranged from 20.4% to 24.2%, yet mortality in the study at the rural hospital in Uganda was only 3.9% [5–8]. The study in Haiti found encephalopathy, supplemental oxygen therapy, and stool microscopy to be predictors of mortality among septic patients [8]. Another study in Uganda found Glasgow Coma Scale (GCS), Karnofsky Performance Scale (KPS), tachypnea, thrombocytopenia, and leukocytosis to be mortality predictors [9].

The optimal treatment of sepsis in low-income countries is unknown. The only randomized controlled trial of sepsis resuscitation in adults in Africa demonstrated harm with fluid resuscitation similar to that considered standard of care in HICs [10]. This may be related to late presentation, underlying infection types that differ from those in HICs, and lack of access to mechanical ventilation after resuscitation [10–12].

We aimed to evaluate the presentation, management, outcomes, and predictors of mortality from sepsis through a retrospective cohort study of patients admitted to two of the three public tertiary hospitals in Rwanda.

## Materials and methods

### Study setting and population

The University Teaching Hospital of Kigali (CHUK) is a public academic tertiary referral hospital in Rwanda’s capital. With approximately 565 total beds, primary facilities at the time of this study include a three-level emergency department, inpatient internal medicine, surgery, pediatrics, obstetrics and gynecology wards, a seven-bed intensive care unit (ICU), and a four-bed high-dependency (step-down) unit.

The University Teaching Hospital of Butare (CHUB) is a public academic referral hospital in the Southern Province of Rwanda. With approximately 490 beds, primary facilities at the time of this study included an emergency department, internal medicine, surgery, pediatrics, and obstetrics and gynecology inpatient wards, and a five-bed ICU.

### Study oversight

The University of Rwanda Institutional Review Board, the Ethics Committee at CHUK, and the Research Ethics Committee at CHUB approved the study. Individual subject consent was waived by the University of Rwanda Institutional Review Board due to determination of minimal level of risk. Data analysis was approved by the Virginia Commonwealth University Institutional Review Board.

### Definitions

Sepsis was defined as suspected or confirmed infection plus two or more of the three quick Sequential [Sepsis-related] Organ Failure Assessment (qSOFA) criteria: respiratory rate ≥ 22, systolic blood pressure ≤ 100, and Glasgow Coma Scale score < 15 [1]. The SOFA-based Sepsis-3 definition for sepsis is not feasible as a definition in this setting, given its requirement for multiple lab values that are not routinely collected in these hospitals. While the qSOFA score was developed as a screening tool for patients who might have poor outcomes, it also reflects signs of inflammation indicative of the dysregulated immune response that defines sepsis [1]. We therefore used qSOFA in our definition of sepsis in this study. Septic shock was defined as sepsis plus either vasopressor support or mean arterial pressure (MAP) ≤ 60 [13]. While serum lactate is included in current definitions of septic shock, it is rarely collected in this setting and thus could not be used. Vital signs on meeting sepsis criteria are defined as the first set of vital signs that met two or more of the qSOFA criteria while there was suspected or confirmed infection documented, or the set of vital signs most proximal to when clinician determination of sepsis was documented. Laboratory values associated with sepsis (white blood cell count, platelet count, creatinine, and bilirubin) were captured if drawn within 24 hours of the time when the patient met sepsis criteria, and the laboratory values most proximal to the time of sepsis were recorded.

Acute respiratory distress syndrome was defined by clinician documentation, as blood gas analysis for determination of PaO2 is not available at CHUB, and is performed infrequently at CHUK. Acute kidney injury was defined by either clinician documentation or by creatinine values in the Kidney Disease: Improving Global Outcomes (KDIGO) criteria: increase in serum creatinine by ≥ 0.3 mg/dl within 48 hours or increase in serum creatinine to ≥ 1.5 times baseline [14]. Coagulopathy was defined as activated partial thromboplastin time (aPPT) > 40 seconds, or international normalized ratio (INR) >1.2, or elevated d-dimer, based on local reference ranges. Acute liver injury was defined as INR ≥ 2.0, ALT ≥10 times the upper limit of normal and bilirubin ≥3.0 mg/dL, and acute liver failure was defined as development of severe acute liver injury with encephalopathy and impaired synthetic function (INR of ≥1.5) in a patient without cirrhosis or preexisting liver disease or by clinician’s documentation [15, 16]. Myocardial infarction was defined by either clinician documentation or as elevated creatine kinase-MB (CK-MB) or elevated troponin (elevations defined based on local reference ranges). Rural and urban districts were defined according to designations from the Rwanda Ministry of Finance and Economic Planning [17].

### Data collection and quality assurance

At CHUK, the study team screened a paper admission and discharge central logbook in which patient medical record number and final diagnosis or diagnoses are recorded. We screened logbook entries with discharge dates between January 1, 2017 and December 31, 2017, and those with a final diagnosis of sepsis, septic shock, or any infection underwent chart review in the medical archives. Once patients met the logbook screening criteria, all paper medical records were accessed in the archive to determine if they met clinical inclusion criteria (presence of sepsis or septic shock as defined below plus age greater than 17 at time of presentation).

CHUB does not have a central admission and discharge logbook. Potential subjects were screened by accessing the central archive and reviewing medical records from the paper medical records partitioned by discharging service and year. From the ICU and from the internal medicine and surgical wards with discharge dates in 2017, we searched for documentation of sepsis, septic shock, or any documented infection in the paper medical records and subsequently determined if the patient met inclusion criteria. We screened all patients discharged from the ICU and a convenience sample of patients discharged from the internal medicine and surgery wards.

A study team comprised of students, residents, and faculty physicians conducted and supervised the data abstraction. All data abstracted was retrospective: vital sign and urine output data in the paper medical record was entered during the time of patient care by either the patient’s nurses or physicians, and the remaining data in the paper medical record was entered by the patient’s physicians. During medical record review, data was entered directly into the web-based Research Electronic Data Capture (REDcap) tool (Nashville, Tennessee) using personal computers (PCs). The initial 60 charts that met study criteria were reviewed in REDCap by DH and MD for quality assurance purposes, and demonstrated some inconsistencies in data collection. The data from these medical records were thus abstracted by DH and MD. Subsequently, the study team had further training through didactic sessions, interactive case studies, and hands-on side-by-side chart review. A standard operating procedure (SOP) document was provided to all data collectors prior to resumption of chart review. For the 121 medical records abstracted by the student data collectors, quality assurance was performed by a resident physician. An audit of 12% of these medical records randomly selected was also performed by WM to ensure consistency with data entered into REDCap.

### Statistical analysis

In-hospital mortality was the primary outcome of interest, as it is an objective outcome criterion for which data was readily available. Continuous data are summarized using medians and interquartile ranges unless otherwise specified. Demographic characteristics, baseline clinical features, and management variables were compared for in-hospital survivors and non-survivors. Continuous data with normal distributions were compared in univariate analysis for model building using equal variance or unequal variance two sample independent t-tests with equality of variances assessed using the Brown-Forsythe test. Comparisons for nonparametric continuous data were made using the Wilcoxon rank-sum test. Categorical variables are summarized with proportions, and differences were compared in univariate analysis for model building using χ^2^ test or Fisher’s exact test. Significance was determined if p-value was less than 0.05.

Multivariable logistic regression of baseline variables was performed to assess for predictors of in-hospital mortality, and predictive variables from this model were used to control for severity of illness when performing an exploratory multivariable logistic regression analysis of potential relationships between management variables and in-hospital mortality. All significant univariates were assessed for interactions with age, sex, hospital, province of residence, and residence in a rural district. Interactions between these variables were included in model selection as potential predictors if p-values were less than 0.05. For both the baseline variable model and management variable models, univariates with p-values less than 0.05 and those with probable clinical significance were selected to be included in a forward stepwise selection methodology, with the p-value threshold set at 0.10. The automated forward stepwise selection method was then performed. Given the large number of significant covariates after performance of forward stepwise selection, those in the model with p-values greater than 0.90 were removed and the logistic regression was performed without these covariates to create the final model.

Multicollinearity was assessed using correlation coefficients and through assessment of parameter sign switching (negative to positive or vice versa), and potential covariates with r > 0.85 and/or those with reversal of sign were assessed and the most clinically relevant covariate was retained in the analysis. We report adjusted odds ratios and 95% confidence intervals of the adjusted odds ratios, likelihood ratio chi square statistics, and p-values for both models. We also performed and report for both models the fit statistics of area under the receiver operating characteristic curve, Hosmer-Lemeshow statistic and p-value, Brier score, and Nagelkerke pseudo R^2^. Statistical analysis was performed with R version 4.0.2 and JMP Pro version 15.

## Results

### Demographic and baseline characteristics

A total of 181 charts met inclusion criteria (100 at CHUK and 81 at CHUB) and were reviewed. Nearly three quarters of our sample were female (72.4%), and the median age was 40 (IQR = 29, 59) (Table 1). Nearly half (48.9%) of the subjects resided in a rural district. The majority (89.0%) of patients were transferred from a district hospital or health center. Of the 113 patients transferred from another health center, we had data on length of stay at the outside facility for 32/113 (28.3%). For those, the median length of stay at these facilities was 2.0 days (IQR = 1.0, 5.5).

**Table 1:** Demographic and baseline characteristics.

	Number of patients with data	Full cohort	Survivors	Non-survivors
	n (% of total cohort)	n (%) or median [IQR]	n (%) or median [IQR]	n (%) or median [IQR]
**Demographic characteristics**				
Sex	181 (100.0)			
Male		50 (27.6)	13 (26.0)	37 (74.0)
Female		131 (72.4)	75 (57.3)	56 (42.7)
Age	178 (98.3)	40 [29,59]	36 [26,57]	44 [31,61]
Province of residence	179 (98.9)			
Southern		79 (44.1)	25 (31.6)	53 (58.2)
Kigali		63 (35.2)	46 (73.0)	17 (18.9)
Western		17 (9.5)	6 (35.3)	11 (64.7)
Eastern		11 (6.1)	5 (45.5)	6 (54.5)
Northern		9 (5.0)	5 (55.6)	4 (44.4)
Reside in urban vs rural district	178 (98.3)			
Urban		91 (51.1)	56 (61.5)	35 (38.5)
Rural		87 (48.9)	30 (34.5)	57 (65.5)
**Presenting characteristics**				
Where patient presented from	127 (70.2)			
District hospital		113 (89.0)	36 (31.9)	77 (68.1)
Home or work		6 (4.7)	4 (66.7)	2 (33.3)
Other		9 (7.1)	7 (77.8)	2 (22.2)
Length of stay at district hospital	32 (17.7)	2.0 [1.0,5.5]	2.0 [1.0,5.5]	2.0 [1.0,6.5]
Hospital presented to	181 (100.0)			
CHUK		100 (55.2)	65 (65.0)	35 (35.0)
CHUB		81 (44.8)	23 (28.4)	58 (71.6)
**Met sepsis criteria at time of hospital admission**	181 (100.0)	108 (59.7)	47 (43.5)	61 (56.5)
**Time from presentation to meeting sepsis criteria**	166 (89.0)	0.0 [0.0,1.25]	0.39 [0.0,1.3]	0.0 [0.0,1.25]
**Vital signs on meeting sepsis criteria**				
Heart rate in beats per minute	175 (96.7)	112 [94,125]	106 [92,120]	120 [104,129]
Systolic blood pressure in mmHg	176 (97.2)	96 [89,118]	96 [91,110]	97 [86,120]
Mean arterial pressure in mmHg	174 (96.1)	71 [64.5,85]	71 [66,80]	73 [61,89]
Respiratory rate in breaths per minute	112 (61.9)	24 [21,28]	23 [20,26]	25 [22,32]
Temperature in degrees Celsius	122 (67.4)	37.1 [36.4,38.0]	37.0 [36.3,38.2]	37.1 [36.5,38.0]
Oxygen saturation	151 (83.4)	95 [92,98]	96 [94,98]	95 [91,98]
Glasgow Coma Scale score	171 (94.5)	15 [11,15]	15 [14,15]	14 [9,15]
**Laboratory results within 24 hours of meeting sepsis criteria**				
White blood cells g/L	152 (84.0)	7.9 [3.9,13.0]	6.6 [3.9.10.7]	8.5 [4.3,13.9]
Platelets g/L	147 (81.2)	171 [68,308]	187 [110,349]	137 [48,296]
Creatinine	127 (70.2)	0.8 [0.5,1.52]	0.85 [0.52,1.37]	0.79 [0.50,1.67]
Positive malaria blood smear	95 (52.5)	21 (22.1)	13 (61.9)	8 (38.1)
Positive *Mycobacterium tuberculosi*s assay	35 (19.3)	5 (14.3)	3 (60.0)	2 (40.0)
**Comorbidities**	181 (100.0)			
HIV		35 (19.3)	24 (68.8)	11 (31.4)
Any other comorbidity^a^		29 (16.0)	13 (44.8)	16 (55.2)
**Source of infection (discharge diagnosis)**^b^	181 (100.0)			
Intra-abdominal		67 (37.0)	22 (32.8)	45 (67.2)
Pulmonary		59 (32.6)	33 (55.9)	26 (44.1)
Skin/wound		21 (11.6)	12 (57.1)	9 (42.9)
Central nervous system		20 (11.0)	5 (25.0)	15 (75.0)
Urine		8 (4.4)	5 (62.5)	3 (37.5)
Bacteremia with unknown source		1 (0.6)	1 (100)	0 (0)
Other		21 (11.6)	14 (66.7)	7 (33.3)

^a^Other comorbidities include diabetes mellitus (n = 11, 6.1%), hypertension (n = 11, 6.1%), hepatitis B or C (n = 5, 2.8%), cancer (n = 3, 1.7%), chronic kidney disease (n = 3, 1.7%), chronic liver disease (n = 3, 1.7%), chronic lung disease (n = 2, 1.1%), and chronic heart disease (n = 1 0.6%).

^b^This is not a mutually exclusive category. One patient may have multiple sources of infection.

One hundred eight subjects (59.7%) met criteria for sepsis at time of hospital admission and 73 met inclusion criteria later in the hospital course. The median time from hospital presentation to meeting criteria for sepsis was 0 days (IQR 0, 1.3). Vital signs were recorded consistently with the exception of respiratory rate and temperature (38.1% and 32.6% of values were missing, respectively) (S1 Table). At time of meeting criteria for sepsis, median heart rate was 112 beats per minute, respiratory rate was 24 breaths per minute, systolic blood pressure was 96 mmHg, temperature was 37.1°C, SpO2 was 95%, and median GCS was 15 (IQR 11, 15) (Table 1). Median white blood cell count, platelet count, and creatinine were within normal limits. Known HIV positive patients represented 19.3% of the sample; 6.1% of the sample had known prior hypertension; 6.1% had diabetes mellitus. Other comorbidities (Hepatitis B or C, malignancy, and chronic kidney, liver, heart, or lung disease) were seen in the subjects at rates less than 5%.

The most common source of infection documented at discharge was intra-abdominal (37.0%) followed by pulmonary (32.6%) (Table 1). Of the 95 patients who received a malaria blood smear, 21 (22.1%; 11.6% of the total cohort) were positive for *Plasmodium* species. Five patients (2.8% of the total cohort) tested positive for *Mycobacterium tuberculosis* either by acid-fast bacilli smear or nucleic acid amplification test. Of the 55 blood cultures obtained, 9 (16.4%) were positive. Sensitivities were available for four of these samples, and antimicrobial resistance was present in three samples (S2 Table). Antimicrobial resistance in urine cultures (n = 4) was present in all samples (S3 Table).

#### Diagnostics and management

Sepsis or septic shock was listed as a diagnosis in the medical record in two-thirds of cases (Table 2). Septic shock criteria were met by 70 (38.7%) subjects. Patients with sepsis or septic shock were most frequently managed in the internal medicine ward. The majority of patients (n = 95, 52.5%) had performance of a malaria blood smear recorded (S4 Table). Blood cultures were drawn in 55 (30.4%) of patients and 36 (19.9%) had a urine culture performed. Twenty-six patients (14.4%) were assessed for tuberculosis by nucleic acid amplification test and 9 (5.0%) were assessed by acid-fast bacilli smear (S4 Table). Ultrasound of any internal organ was performed in 45.3% of patients and 34.8% of patients received a chest radiograph (S4 Table).

**Table 2:** Management.

	Number of patients with data	Full cohort	Survivors	Non-survivors
	n (% of total cohort)	n (%) or median [IQR]	n (%) or median [IQR]	n (%) or median [IQR]
**Sepsis named in the patient’s chart**	180 (99.4)	120 (66.7)	43 (35.8)	77 (64.2)
**Primary location of sepsis management**	179 (98.9)			
Medicine ward		77 (43.0)	58 (75.3)	19 (20.7)
ICU		57 (31.8)	12 (21.1)	45 (48.9)
A&E		27 (15.1)	9 (33.3)	18 (19.6)
Surgery Ward		14 (7.8)	8 (57.1)	6 (42.9)
OB/GYN Ward		4 (2.2)	0 (0)	4 (100)
**Intravenous fluid resuscitation**	123 (68.0)			
Volume (L) received in first 8 hours		1.0 [0.5,2.0]	1.0 [0.5,2.0]	2.0 [1.0,2.7]
Volume (L) received in first 24 hours		2.0 [1.0,4.0]	1.5 [1.0,2.8]	2.5 [1.5,4.6]
**Antimicrobials**^a^	181 (100.0)			
At least one antimicrobial administered		170 (93.9)	84 (49.4)	86 (50.6)
Ceftriaxone		108 (59.7)	52 (48.1)	56 (51.9)
Metronidazole		76 (42.0)	24 (31.6)	52 (68.4)
Cefotaxime		15 (8.3)	2 (13.3)	13 (86.7)
Artesunate		13 (7.2)	9 (169.2)	4 (30.8)
Doxycycline		10 (5.5)	7 (70.0)	3 (30.0)
**Glucocorticoid therapy**	181 (100.0)	14 (7.7)	3 (21.4)	11 (78.6)
**Vasopressors**	181 (100.0)			
At least one vasopressor administered		58 (32.0)	4 (6.9)	54 (93.1)
Vasopressors administered^b^				
Epinephrine		38 (21.0)	4 (10.5)	34 (89.5)
Dopamine		23 (12.7)	0 (0)	23 (100)
Norepinephrine		13 (7.2)	0 (0)	13 (100)
**Mechanical ventilation**	181 (100.0)			
Received mechanical ventilation		84 (46.4)	14 (16.7)	70 (83.3)
**Duration of vasopressors and mechanical ventilation**				
Duration of vasopressors in days	76 (42.0)	0.7 [0.3,2.1]	-	-
Duration of mechanical ventilation in days	69 (38.1)	2.4 [0.8,7.2]	3.0 [0.8,16.8]	2.0 [0.8,6.9]
**Procedures performed**	181 (100.0)			
Intubation		80 (44.2)	12 (15.0)	68 (85.0)
Exploratory laparotomy		43 (23.8)	9 (20.9)	34 (79.1)
Abscess drainage		9 (5.0)	2 (22.2)	7 (77.8)
Central line		9 (5.0)	1 (11.1)	8 (88.9)
Wound debridement		9 (5.0)	3 (33.3)	6 (66.7)
Bronchoscopy		3 (1.7)	2 (66.7)	1 (33.3)
Other		12 (6.6)		

^a^for full list, see S5 Table.

^b^multiple vasopressors can be administered to the same patient

The vast majority of patients (92.7%) received intravenous fluid resuscitation within 8 hours and 96.8% received fluid resuscitation within 24 hours (Table 2). The median volume of intravenous fluid administered in the first 8 hours after meeting sepsis criteria was 1.0 L (IQR = 0.5, 2.0 L), and in the first 24 hours the median intravenous fluid received was 2.0 L (IQR = 1.0, 4.0 L). Nearly all patients (94.0%) received at least one antimicrobial, and the most frequently administered antimicrobials were ceftriaxone and metronidazole (S5 Table). Antimicrobials with broader spectrums of activity were frequently added later in the hospital course (S2 Table). Exploratory laparotomy was performed on 23.8% of subjects (Table 2).

Vasopressors were administered in 58 patients (32.0%) and the most frequently administered vasopressor was epinephrine followed by dopamine (Table 2). A total of 46.4% of patients with sepsis received mechanical ventilation. The median duration of vasopressors and mechanical ventilation was 0.7 days (IQR 0.3, 2.1) and 2.4 days (IQR 0.8, 7.2), respectively. Glucocorticoids were administered in 7.7% of cases, and 9 patients (5.0%) underwent central venous catheter placement.

### Outcomes

Acute kidney injury was the most common complication (19.3%), followed by acute respiratory distress syndrome (9.9%) (Table 3). The median length of stay in the ICU was 2.3 days and the median length of stay in the internal medicine and surgery wards were 9.4 days and 6.0 days, respectively. The overall median length of stay in the referral hospital was 9.5 days. The in-hospital mortality rate for sepsis or septic shock in this study was 51.4%. Mortality for septic shock was 82.9% and for sepsis without septic shock was 31.5%.

**Table 3:** Outcomes.

	Number of patients with data	Full cohort	Survivors	Non-survivors
	n (% of total cohort)	n (%) or median [IQR]	n (%) or median [IQR]	n (%) or median [IQR]
**Complications**	181 (100.0)			
Acute kidney injury		35 (19.3)	7 (20.0)	28 (80.0)
Acute respiratory distress syndrome (ARDS)		18 (9.9)	4 (22.2)	14 (77.8)
Acute liver injury or liver failure		2 (1.1)	1 (50.0)	1 (50.0)
Coagulopathy		1 (0.6)	1 (100)	0 (0)
Myocardial infarction		0 (0)	0 (0)	0 (0)
**Length of stay by service in days**				
Emergency department	104 (57.5)	0.6 [0.2,1.1]	0.7 [0.2,1.0]	0.6 [0.2,1.3]
Medicine ward	77 (42.5)	9.4 [4.5,15.9]	10.4 [5.7,18.1]	7.6 [3.0,9.6]
Surgery ward	29 (16.0)	6.0 [1.0,21.6]	18.7 [2.0,30.4]	1.2 [0.9,12.2]
Obstetrics and gynecology ward	14 (7.7)	0.4 [0.2,6.8]	3.2 [0.3,8.3]	0.4 [0.1,4.6]
Intensive care unit	80 (44.2)	2.3 [0.3,7.0]	6.0 [1.0,23.4]	1.9 [0.5,6.7]
**Length of stay at referral hospital**	173 (95.6)	9.5 [4.1,19.9]	12.7 [6.9,23.8]	6.7 [1.9,11.8]
**Disposition**	181 (100.0)			
Died		93 (51.4)		
Sepsis (without shock) and died		35 (31.5)		
Sepsis with shock and died		58 (82.9)		
Discharge to home		73 (40.8)		
Discharge to district hospital		12 (6.6)		
Eloped from hospital		1 (0.6)		

#### Predictors of mortality

On univariate analysis, presentation characteristics associated with in-hospital mortality were male sex, age, province of residence, residing in a rural district, transfer from a district hospital, presentation to CHUB, high respiratory rate when meeting inclusion criteria, lower GCS score when meeting inclusion criteria, lower platelet count, HIV positive status, and intra-abdominal or central nervous system source of infection. There were no significant interactions between assessed covariates. Baseline characteristics at the time of meeting sepsis criteria with statistical significance that predicted mortality on multivariable logistic regression were one breath per minute increase in respiratory rate (adjusted odds ratio (aOR) = 1.10, 95% CI = 1.01–1.21, p-value = 0.037), one point decrease in GCS (aOR = 0.793, 95% CI = 0.621–0.968, p-value = 0.021), and known HIV+ status (aOR = 7.02, 95% CI = 1.15–55.8, p-value = 0.034) (S6 Table). The area under the receiver operating characteristic curve for this model was 0.861 (95% CI: 0.787–0.934). Additional fit statistics include Hosmer-Lemeshow chi-square statistic of 3.0662 (p-value = 0.930), Brier Score = 0.150, and Nagelkerke pseudo R^2^ = 0.513.

Management characteristics on univariate analysis associated with higher mortality included higher volume of fluid resuscitation in the first eight hours after sepsis presentation, management in the intensive care unit, urine output not recorded by nursing staff, receiving metronidazole or cefotaxime, receiving steroids, receiving vasopressor therapy, receiving mechanical ventilation, and undergoing intubation, exploratory laparotomy, or central venous catheter placement (Table 2). No significant interactions were found between assessed covariates. Nearly all (93.1%) patients who received at least one vasopressor died and the vast majority (83.3%) of those who received mechanical ventilation died. Upon controlling for age, sex, and the predictors identified in the presentation characteristics model (illness severity indicators of respiratory rate and GCS, and known HIV+ status) the management factors with statistical significance associated with mortality on multivariable logistic regression were administration of vasopressors (aOR = 7.46, 95% CI = 1.69–40.2, p-value = 0.007) and mechanical ventilation (aOR = 6.65, 95% CI = 1.04–51.6, p-value = 0.046) (S7 Table). The area under the receiver operating characteristic curve for this model was 0.895 (95% CI: 0.829–0.961). Additional fit statistics for this model include a Hosmer-Lemeshow chi-square statistic of 7.943 (p-value = 0.439), Brier score = 0.127, and Nagelkerke pseudo R^2^ = 0.569.

## Discussion

We examined presentation, management, and outcomes of sepsis and septic shock at two tertiary referral hospitals in Rwanda. Mortality from sepsis without shock was 31.5%, and mortality from septic shock was 82.9%. The most common source of infection was intra-abdominal. Intravenous fluid resuscitation and antimicrobials were administered in most cases. Baseline characteristics at time of meeting sepsis criteria predicting mortality included respiratory rate, GCS score, and HIV+ status. When adjusting for these illness severity factors, management predictors of mortality were administration of vasopressors and mechanical ventilation.

Intravenous fluid resuscitation, antimicrobial administration, and source control are key aspects of sepsis management. In our sample, 93% received intravenous fluid resuscitation within 8 hours and the median volume received within this time frame was 1.0 L. The evidence for optimal standards of intravenous fluid resuscitation in sepsis in sub-Saharan Africa has been mixed [9–12]. In the only randomized controlled trial assessing intravenous fluid resuscitation in adults with sepsis in sub-Saharan Africa, Andrews et al. [10] found higher mortality in patients who received a greater volume of intravenous fluid resuscitation (3.5 L compared to 2.0 L within the first 6 hours after presentation). The Fluid Expansion As Supportive Therapy (FEAST) trial in African children revealed similar findings, as patients who received intravenous fluid bolus experienced higher mortality [18]. The most appropriate volume of fluid resuscitation in patients with sepsis remains unclear.

The vast majority of our sample (94.0%) received antimicrobial therapy. The most frequent antibiotic administered was ceftriaxone, and while we only had culture data from 8 patients in this study, all isolates tested for ceftriaxone were resistant to it. Our findings are similar to a 2018 study of peritonitis in Rwanda, in which 95.8% of patients with infectious peritonitis (95% meeting criteria for sepsis or severe sepsis) received antibiotics and the most frequently prescribed antibiotics were third generation cephalosporins (90% of cases) and metronidazole (85% of cases) [19]. Cephalosporin resistance was also high in that study as only one of seven isolates was sensitive to ceftriaxone. High rates of cephalosporin resistance in Rwanda has been demonstrated in several other studies as well [20–23]. Significantly more patients in our study received antimicrobials besides ceftriaxone and metronidazole later in their hospital course, presumably on clinical deterioration. Regarding source control, we collected data on intra-abdominal infections, and 43 out of 67 patients (64.2%) with an intra-abdominal source of infection underwent exploratory laparotomy.

Our findings of GCS, respiratory rate, and HIV+ status as significant baseline characteristics at time of meeting sepsis criteria predicting mortality have some overlap with Moore et al.’s [24] Universal Vital Assessment (UVA) score and Riviello et al.’s [4] Rwanda Mortality Probability Model (R-MPM). The UVA score, developed from data from 6 countries in sub-Saharan Africa, serves to predict mortality of patients in resource-limited settings admitted to the hospital for any reason. In addition to our above predictors that align with the UVA score, the score also includes temperature, heart rate, and systolic blood pressure. The R-MPM, based on data from patients admitted to the ICU, employs the mortality predictors of age, suspected or confirmed infection within 24 hours of ICU admission, hypotension or shock as a reason for ICU admission, Glasgow Coma Scale score (aligns with our findings) at ICU admission, and heart rate at ICU admission. In another study on peritonitis in Rwanda in which 83% of patients had sepsis or severe sepsis, Ndayizeye et al. [25] found that predictors of mortality included unplanned reoperation, vasopressor use (aligns with our findings), abnormal white blood cell count, ICU admission, and American Society of Anesthesiologist score of ≥ 3.

We found mechanical ventilation and administration of vasopressors to be the only management predictors of mortality. While it is possible that these management modalities do result in worse outcomes given risks of harm with these interventions, it is more likely that the use of mechanical ventilation or vasopressors are indicators of severity of illness that were not captured by our three-variable model. Further study is needed.

Our study had several limitations. First, we defined sepsis as suspected or confirmed infection plus two or more of the three qSOFA criteria (respiratory rate ≥ 22, systolic blood pressure ≤ 100, and Glasgow Coma Scale score < 15) [1]. The Sepsis-3 task force defined sepsis as “life-threatening organ dysfunction caused by a dysregulated host response to infection.” They suggested that the definition could be operationalized as an increase in SOFA score of 2 or more points, due to infection. They further suggested that the qSOFA criteria could be used to identify patients with a high likelihood of a poor outcome, particularly in out of hospital, emergency department, and ward settings [1]. Both operationalizations of the conceptual definition correlated with mortality. While the increase in SOFA score of two points is more commonly used to operationalize the definition for research, it was not possible in our cohort given that PaO2 and bilirubin, two key components of SOFA, are almost never available in this setting. We therefore used the qSOFA criteria to operationalize the definition of sepsis. It is not clear in what direction this might bias our results, but our population may be different than other cohorts that are defined using the SOFA score. We were also unable to include lactate in our definition of septic shock; again, this may mean that our cohort is somewhat different than others that define septic shock using both blood pressure and lactate. It is not clear in what direction this would bias our results.

Second, both our methods, as well as limitations in clinical documentation, led to a selection bias toward the inclusion of ICU patients over ward patients. While we screened all patients at CHUK discharged in 2017 for our inclusion criteria, at CHUB we screened all patients discharged in 2017 from the ICU, but only screened a portion of patients discharged from the internal medicine and surgery wards due to our resource limitations. This results in missing cases of sepsis at CHUB and a selection bias at CHUB toward patients discharged from the ICU. At both hospitals, vital signs are recorded with greater consistency in the ICU than in the wards, which may have led to additional selection bias toward ICU patients, since vital signs are included in the inclusion criteria. Finally, our inclusion criterion of two out of three qSOFA criteria, as opposed to one out of three criteria, may have resulted in bias toward more critically ill patients since every one point increase in qSOFA score is associated with higher mortality [26, 27]. We chose to use two out of three qSOFA criteria to achieve higher specificity [28, 29]. All of these factors lead to a selection bias that favors ICU patients being included in our study. This means that outcomes may be worse than might be expected from a comprehensive sampling of all hospital patients with sepsis.

Third, the limitations of clinical documentation for use in research led to an undercounting of sepsis cases, as well as missing data for the participants included. The CHUK central admission and discharge logbook lists final diagnosis or diagnoses as free text entries without standardization, and often with only primary diagnosis listed. It is possible that patients could have an infection or sepsis as one of their diagnoses, but that this would not be listed in the logbook. This may have led to fewer cases of sepsis at CHUK being captured. Indeed, previous studies have found higher proportions of hospitalized patients with a sepsis diagnosis [7, 30, 31], suggesting our method of screening may have missed patients with sepsis. Additionally, as this was a retrospective study, we were limited to data that was available in the medical records. Missing data rates ranged from 0% to 85.6%. This may have led to lower detection rates of sepsis since the three variables in the qSOFA score used for defining sepsis here include variables that are inconsistently recorded. This study cannot speak to the incidence or prevalence of sepsis at these facilities, given that our screening methods and inclusion criteria are likely to have missed cases of sepsis.

Missing values also mean that data on the included patients is incomplete in some cases. Laboratory values and imaging results are recorded inconsistently in physician documentation or are available on slips of paper which may be lost. Additionally, cardiac enzymes, liver and coagulation profiles, and arterial blood gasses are rarely ordered and thus our rates of myocardial infarction, liver failure, coagulopathy, and ARDS are almost certainly lower than actual rates. Patients may have had underlying comorbidities that were not documented in the medical record.

Fourth, we were limited in our ability to define septic shock using established criteria since serum lactate level is rarely available in our setting, and vasopressors are not consistently available to all patients given resource constraints. We were able to define septic shock only based on whether a vasopressor was administered, or whether a patient had hypotension. Fifth, we were unable to determine the timing of antimicrobial administration as the precise time is infrequently recorded.

Finally, while we attempted to control for severity of illness in the management mortality predictors model by including baseline characteristics that predicted mortality, we found nonetheless that receiving mechanical ventilation or vasopressor therapy remained as mortality predictors in the final model. The severity of illness scores are imperfect. The predictive value of mechanical ventilation and vasopressor therapy likely reflect the fact that more critically ill patients require these interventions, not that these interventions themselves are a cause of mortality.

The limitations of our study themselves suggest potential areas of quality improvement in sepsis care: monitoring and documentation of vital signs, as well as recognition of sepsis. Respiratory rate was missing in 38% of charts, and temperature in 33%. One-third of the subjects we identified as having sepsis did not have a sepsis diagnosis named in the medical record.

Future studies would greatly benefit from prospective data collection to document other aspects of care that may not have been recorded, to determine precise antimicrobial administration time and antimicrobial resistance patterns, and to assess potential delays in access to care and delays in operative procedures for source control, as these have been shown to be high and likely predict poor outcomes [32].

## Conclusion

Sepsis and septic shock remain understudied in sub-Saharan Africa. Sepsis and septic shock mortality were high in our sample of patients at two tertiary referral hospitals. The vast majority of patients received therapy with antimicrobials and intravenous fluid resuscitation. Baseline characteristics that predicted mortality in our sample were GCS, respiratory rate, and HIV+ status. Future work should focus on optimal targets for intravenous fluid resuscitation, antimicrobial resistance and timing of administration, and appropriate use of vasopressors and mechanical ventilation.

## Supporting information

S1 Table Missing values. (DOC)

S2 Table Blood culture sensitivities. (DOCX)

S3 Table Urine culture sensitivities. (DOCX)

S4 Table Diagnostics. (DOCX)

S5 Table Antimicrobials administered. (DOCX)

S6 Table Presentation characteristics predictors of in-hospital mortality. (DOCX)

S7 Table Exploratory management predictors of in-hospital mortality controlling for severity of illness. (DOCX)
