# Supplementary material for: Sepsis in two hospitals in Rwanda: A retrospective cohort study of presentation, management, outcomes, and predictors of mortality
Source: PLoS One. 2021 May 26;16(5):e0251321. doi: 10.1371/journal.pone.0251321 (PMC8153478; doi:10.1371/journal.pone.0251321)
Supplement: S1 Table — (DOC) [file pone.0251321.s001.doc]

S1 Table. Missing values.

|  | **Missing values**  **n (%)** |
| --- | --- |
| **Demographics and baseline characteristics** |  |
| Demographic characteristics | 0 (0) |
| Sex | 3 (1.7) |
| Age | 3 (1.7) |
| Province of residence | 2 (1.1) |
| District of residence | 3 (1.7) |
|  |  |
| Presenting characteristics |  |
| Where patient presented from | 54 (29.8) |
| Length of stay at district hospital^a^ | 81 (71.7) |
| Mode of transportation to hospital | 155 (85.6) |
| Hospital presented to | 0 (0) |
|  |  |
| Vital signs on meeting sepsis criteria |  |
| Heart rate in beats per minute | 6 (3.3) |
| Systolic blood pressure in mmHg | 5 (2.8) |
| Mean arterial pressure in mmHg | 7 (3.9) |
| Respiratory rate in breaths per minute | 69 (38.1) |
| Temperature in degrees Celsius | 59 (32.6) |
| Oxygen saturation | 30 (16.6) |
| Glasgow Coma Scale score | 10 (5.5) |
|  |  |
| Laboratory values on meeting sepsis criteria |  |
| White blood cells g/L | 29 (16.0) |
| Platelets g/L | 34 (18.8) |
| Creatinine | 54 (29.8) |
| Source of infection | 0 (0) |
| Culture antimicrobial sensitivities |  |
| Blood culture^b^ | 5 (55.6) |
| Urine culture^c^ | 1 (20) |
| **Management** |  |
| Sepsis or septic shock named in the patient's medical record | 1 (0.6) |
| Primary location of sepsis management | 2 (1.1) |
|  |  |
| **Outcomes** |  |
| Length of stay by service in days | 7 (3.9) |
| Length of stay at referral hospital | 8 (4.4) |

**^a^Percent of patients presenting from district hospital**

**^b^Percent of positive blood cultures**

**^c^Percent of positive urine cultures**
