# Supplementary material for: Sepsis in two hospitals in Rwanda: A retrospective cohort study of presentation, management, outcomes, and predictors of mortality
Source: PLoS One. 2021 May 26;16(5):e0251321. doi: 10.1371/journal.pone.0251321 (PMC8153478; doi:10.1371/journal.pone.0251321)
Supplement: S2 Table — (DOCX) [file pone.0251321.s002.docx]

**S2 Table. Blood culture sensitivities.**

|  | **Ceftriaxone** | **Cefotaxime** | **Ciprofloxacin** | **Levofloxacin** | **Ceftazidime** |
| --- | --- | --- | --- | --- | --- |
| *Escherichia coli* |  |  | S | S |  |
| *Klebsiella pneumoniae* | R | R | S |  | S |
| *Salmonella typhii* | R |  | R |  |  |
| *Staphylococcus aureus* |  |  | R |  |  |

R = resistant, S = sensitive, blank = sample not tested for sensitivity to that antibiotic. n = 1 for each species.
