# Supplementary material for: Sepsis in two hospitals in Rwanda: A retrospective cohort study of presentation, management, outcomes, and predictors of mortality
Source: PLoS One. 2021 May 26;16(5):e0251321. doi: 10.1371/journal.pone.0251321 (PMC8153478; doi:10.1371/journal.pone.0251321)
Supplement: S3 Table — (DOCX) [file pone.0251321.s003.docx]

**S3 Table. Urine culture sensitivities**

|  | **Ceftriaxone** | **Cefotaxime** | **Ciprofloxacin** | **Trimethoprim-sulfamethoxazole** | **Gentamicin** | **Imipenem** |
| --- | --- | --- | --- | --- | --- | --- |
| *Escherichia coli* specimen 1 |  |  |  |  | R |  |
| *Escherichia coli* specimen 2 | R | R | R | R | R | S |
| *Escherichia coli* specimen 3 |  | R | R | R |  |  |
| *Acinetobacter spp.* specimen | R |  |  | R | R |  |

R = resistant, S = sensitive, blank = sample not tested for sensitivity to that antibiotic
