# Supplementary material for: Sepsis in two hospitals in Rwanda: A retrospective cohort study of presentation, management, outcomes, and predictors of mortality
Source: PLoS One. 2021 May 26;16(5):e0251321. doi: 10.1371/journal.pone.0251321 (PMC8153478; doi:10.1371/journal.pone.0251321)
Supplement: S4 Table — (DOCX) [file pone.0251321.s004.docx]

**S4 Table. Diagnostics.**

|  | | **Full cohort** | | **Survivors** | | | **Non-survivors** |
| --- | --- | --- | --- | --- | --- | --- | --- |
|  | | n (%) or median [IQR] | | n (%) or median [IQR] | | | n (%) or median [IQR] |
| **Laboratory diagnostics** | |  | |  | | |  |
| Serum lactate assay | | 0 (0) | |  | | |  |
| Malaria blood smear | | 95 (52.5) | | 56 (58.9) | | | 39 (41.1) |
| Nucleic acid amplification test for tuberculosis | | 26 (14.4) | | 22 (84.6) | | | 4 (15.4) |
| Acid fast bacilli smear | | 9 (5.0) | | 6 (66.7) | | | 3 (33.3) |
| Blood culture | | 55 (30.4) | | 25 (45.5) | | | 30 (54.5) |
| Urine culture | | 36 (19.9) | | 22 (61.1) | | | 14 (38.9) |
| Wound culture | | 9 (5.0) | | 5 (55.6) | | | 4 (44.4) |
| Tracheal aspirate culture | | 9 (5.0) | | 5 (55.6) | | | 4 (44.4) |
| Diagnostic lumbar puncture | | 18 (9.9) | | 7 (38.9) | | | 11 (61.1) |
| Diagnostic thoracentesis | | 7 (3.9) | | 6 (85.7) | | | 1 (14.3) |
| Diagnostic paracentesis | | 2 (1.1) | | 1 (50.0) | | | 1 (50.0) |
| **Urine output tracked** | | 35 (19.3) | | 5 (14.3) | | | 30 (85.7) |
| **Imaging performed** | |  | |  | | |  |
| Ultrasound | | 82 (45.3) | | 47 (57.3) | | | 35 (42.7) |
| Chest x-ray | | 63 (34.8) | | 27 (42.9) | | | 36 (57.1) |
| Computed tomography (CT) | | 17 (9.4) | | 13 (76.5) | | | 4 (23.5) |
| X-ray other than chest | | 17 (9.4) | | 3 (17.6) | | | 14 (82.4) |
| Magnetic resonance imaging (MRI) | | 0 (0) | |  | | |  |
|  | |  | |  | | |  |
|  |  | |  | |  |  |  |
