# Supplementary material for: Sepsis in two hospitals in Rwanda: A retrospective cohort study of presentation, management, outcomes, and predictors of mortality
Source: PLoS One. 2021 May 26;16(5):e0251321. doi: 10.1371/journal.pone.0251321 (PMC8153478; doi:10.1371/journal.pone.0251321)
Supplement: S5 Table — (DOCX) [file pone.0251321.s005.docx]

**S5 Table. Antimicrobials administered.**

|  | **Administered upon meeting sepsis criteria (n = 181)**  n (%) | **Administered at any time during hospital course (n = 181)**  **n (%)** |
| --- | --- | --- |
| **Antimicrobial** |  |  |
| Antibiotic |  |  |
| Ceftriaxone | 108 (59.7) | 145 (80.1) |
| Metronidazole | 76 (42.0) | 110 (60.8) |
| Cefotaxime | 15 (8.3) | 27 (14.9) |
| Doxycycline | 10 (5.5) | 45 (24.9) |
| Ciprofloxacin | 7 (3.9) | 20 (11.0) |
| Trimethoprim-sulfamethoxazole | 4 (2.2) | 10 (5.5) |
| Imipenem | 3 (1.7) | 13 (7.2) |
| Clindamycin | 2 (1.1) | 5 (2.8) |
| Meropenem | 1 (0.6) | 5 (2.8) |
| Ampicillin | 1 (0.6) | 4 (2.2) |
| Gentamicin | 1 (0.6) | 5 (2.8) |
| Erythromycin | 1 (0.6) | 8 (4.4) |
| Vancomycin | 1 (0.6) | 3 (1.7) |
| Levofloxacin | 0 (0) | 3 (1.7) |
| Azithromycin | 0 (0) | 2 (1.1) |
| Ceftazidime | 0 (0) | 1 (0.6) |
| Antiviral |  |  |
| Acyclovir | 3 (1.7) | 5 (2.8) |
| Antifungal |  |  |
| Fluconazole | 1 (0.6) | 7 (3.9) |
| Amphotericin | 0 (0) | 2 (1.1) |
| Antiparasitic |  |  |
| Artesunate | 13 (7.2) | 21 (11.6) |
| Artemether / lumefantrine | 0 (0) | 15 (8.3) |
| Other | 12 (6.6) | 43 (23.8) |
