# Supplementary material for: Sepsis in two hospitals in Rwanda: A retrospective cohort study of presentation, management, outcomes, and predictors of mortality
Source: PLoS One. 2021 May 26;16(5):e0251321. doi: 10.1371/journal.pone.0251321 (PMC8153478; doi:10.1371/journal.pone.0251321)
Supplement: S6 Table — (DOCX) [file pone.0251321.s006.docx]

**S6 Table. Presentation characteristics predictors of in-hospital mortality.**

|  | **Adjusted Odds Ratio** | **Adjusted Odds Ratio 95% CI** | **p-value** |
| --- | --- | --- | --- |
| Age | 1.03 | 0.992 - 1.07 | 0.126 |
| Sex (female) | 0.267 | 0.043 - 1.33 | 0.109 |
| Hospital (CHUB) | 2.07 | 0.348 - 12.7 | 0.420 |
| Province of residence (Kigali-all others) | 0.271 | 0.052 - 1.27 | 0.098 |
| Transfer from district hospital | 3.08 | 0.831 - 12.1 | 0.093 |
| Heart rate (beats per minute) | 1.02 | 0.990 - 1.05 | 0.232 |
| Systolic blood pressure (mmHg) | 1.01 | 0.983 - 1.03 | 0.637 |
| Respiratory rate (breaths per minute) | 1.10 | 1.01 - 1.22 | 0.037 |
| Glasgow Coma Scale score | 0.793 | 0.621 - 0.968 | 0.021 |
| Known HIV+ status | 7.02 | 1.15. – 55.8 | 0.034 |
| *Overall model evaluation* | **χ²** | **p-value** | **AUC** |
|  | 44.41 | <0.001 | 0.861 (95% CI: 0.787-0.934) |
