# Supplementary material for: Sepsis in two hospitals in Rwanda: A retrospective cohort study of presentation, management, outcomes, and predictors of mortality
Source: PLoS One. 2021 May 26;16(5):e0251321. doi: 10.1371/journal.pone.0251321 (PMC8153478; doi:10.1371/journal.pone.0251321)
Supplement: S7 Table — (DOCX) [file pone.0251321.s007.docx]

**S7 Table. Exploratory management predictors of in-hospital mortality controlling for severity of illness.**

|  | **Adjusted Odds Ratio** | **Adjusted Odds Ratio 95% CI** | **p-value** |
| --- | --- | --- | --- |
| Management primarily in intensive care unit | 0.362 | 0.051 – 1.97 | 0.248 |
| Underwent exploratory laparotomy | 2.29 | 0.479 - 11.6 | 0.297 |
| Underwent central line placement | 4.14 | 0.363 - 103.5 | 0.262 |
| Received mechanical ventilation | 6.65 | 1.04 – 51.6 | 0.046 |
| Received vasopressor therapy | 7.46 | 1.69 - 40.2 | 0.007 |
| *Overall model evaluation* | **χ²** | **p-value** | **AUC** |
|  | 53.22 | <0.001 | 0.895 (95% CI: 0.829-0.961) |
